# Multigenerational analysis of sex-specific phenotypic differences at midgestation caused by abnormal folate metabolism

**DOI:** 10.1093/eep/dvx014

**Published:** 2017-11-03

**Authors:** Nisha Padmanabhan, Joanna Rakoczy, Monika Kondratowicz, Katerina Menelaou, Georgina E T Blake, Erica D Watson

**Affiliations:** 1 Department of Physiology, Development and Neuroscience, University of Cambridge, Cambridge, UK; 2 Centre for Trophoblast Research, University of Cambridge, Cambridge, UK

**Keywords:** folic acid, MTRR, sexual dimorphism, developmental phenotypes, intrauterine growth restriction, congenital malformations, placenta, transgenerational epigenetic inheritance

## Abstract

The exposure to adverse environmental conditions (e.g. poor nutrition) may lead to increased disease risk in an individual and their descendants. In some cases, the results may be sexually dimorphic. A range of phenotypes has been associated with deficiency in or defective metabolism of the vitamin folate. However, the molecular mechanism linking folate metabolism to development is still not well defined nor is it clear whether phenotypes are sex-specific. The enzyme methionine synthase reductase (MTRR) is required for the progression of folate metabolism and the utilization of methyl groups from the folate cycle. Previously, we showed that the hypomorphic *Mtrr^gt^* mutation in mice results in metabolic disruption, epigenetic instability, and a wide spectrum of developmental phenotypes (e.g. growth defects, congenital malformations) at midgestation that appear in subsequent wild-type generations. This transgenerational effect only occurs through the maternal lineage. Here, we explore whether the phenotypes that result from either intrinsic or ancestral *Mtrr* deficiency are sexually dimorphic. We found that no sexual dimorphism is apparent in either situation when the phenotypes were broadly or specifically defined. However, when we focused on the group of phenotypically normal conceptuses derived from maternal grandparental *Mtrr* deficiency, we observed an apparent increase in placental efficiency in each subsequent generation leading to F4 generation female embryos that weigh more than controls. These data suggest that ancestral abnormal folate metabolism may lead to male grandprogeny that are less able to adapt or female grandprogeny that are programmed to become more sensitive to folate availability in subsequent generations.

## Introduction

The influence of the environment on disease risk is a concept that challenges the view that genetic factors are the primary contributors to noncommunicable diseases [1]. The effects are far-reaching since exposure to environmental insults (e.g. poor diet, toxins, psychological stress, etc.) resulting in disease may occur in early postnatal life, during *in utero* development [2], or in the germ cells prior to conception [3–5]. Mounting evidence demonstrates that disease risk can be inherited by subsequent generations even if they were not exposed to the insult themselves [6–9]. The mechanism, which is complex and currently not well understood, likely involves the disruption of epigenetic factors including DNA methylation, histone modifications, and/or noncoding RNAs that influence gene expression, and thus alter cell function and phenotype [10].

A sex-specific response by offspring to an ancestral environmental insult is a further mechanistic intricacy that will shape our understanding of multigenerational effects. Several studies have shown that female and male offspring display different phenotypes following the same environmental insult *in utero* with respect to timing, onset and severity of outcomes (reviewed by Ref. [2]). For instance, prenatal smoking habits of paternal or maternal grandmothers lead to transgenerational differences in weight, height, and fitness of their grandchildren in a sex-dependent manner [11], though the mechanism is unclear. Perhaps epigenetic alterations, which may occur stochastically in response to an insult to germ cells or early embryos, may affect transcription in a sex-specific manner leading to dimorphic phenotypes. Under normal conditions, transcriptional differences between sexes are found when the embryonic genome is activated [12], which occurs well before gonadal differentiation. Indeed, the normal rate of fetal and placental growth is dependent on sex [13].

Folate (also known as folic acid) is a vitamin that acts as a carrier of methyl groups destined for the methylation of many downstream targets including DNA, RNA, and proteins [14–16], thus strongly implicating its metabolism in epigenetic regulation. In mammals, MTRR (methionine synthase reductase, also known as MSR) is a key enzyme responsible for the progression of folate and methionine cycles. It stimulates the enzymatic activity of methionine synthase through the reductive methylation of its vitamin B12 cofactor [17] leading to methionine synthesis, the precursor of the methyl donor S-adenosylmethionine. Disruption of the folate cycle by the hypomorphic *Mtrr^gt^* mutation causes metabolic dysregulation (e.g. increased homocysteine and 5-methyltetrahyrofolate), epigenetic instability (e.g. global DNA hypomethylation and locus-specific DNA hypo- and hypermethylation), and a wide spectrum of developmental phenotypes at midgestation, such as growth defects (e.g. growth restriction and enhancement, developmental delay) and congenital malformations (e.g. neural tube, heart, placental defects) [8, 18]. Many of these phenotypes are similar to those observed in cases of dietary folate deficiency [19, 20].

Remarkably, we recently showed that a transgenerational effect on development occurs when either maternal grandparent (F0 generation) is a carrier for the *Mtrr^gt^* mutation [8]. In this case, their wild-type grandprogeny (F2 generation) displayed similar phenotypes as *Mtrr^gt/gt^* conceptuses (derived from a different pedigree), as well as alterations in DNA methylation patterns associated with gene misexpression [8]. Moreover, the two main phenotypic classes (i.e. growth defects and congenital malformations) resulting from maternal grandparental *Mtrr* deficiency are the consequences of separable epigenetic mechanisms as determined by embryo transfer experiments [8]. The growth phenotypes, which did not persist at significant frequencies past the F2 generation, are the result of an atypical uterine environment in the wild-type F1 generation [8]. Conversely, the congenital malformations result from inheritance of a yet-to-be determined epigenetic factor via the germ line and were shown to appear for at least four wild-type generations (i.e. the F4 generation) [8]. This indicates that the *Mtrr^gt^* mouse line is a model of transgenerational epigenetic inheritance.

Dietary folate deficiency and polymorphisms in folate metabolic enzymes may cause sex-specific effects in some cases in late gestation, early postnatal life, or in adulthood [21–25]. The importance of folate during the preconception period and early pregnancy is widely reported [26]. Therefore, assessing whether abnormal folate metabolism leads to sex-specific effects at an earlier stage of development when severe, potentially lethal phenotypes are present will determine whether there is a sexual bias at a critical developmental time point. In this study, we sought to determine whether the developmental phenotypes characterized in the *Mtrr^gt^* mouse model are sexually dimorphic at midgestation (i.e. embryonic day [E] 10.5). Ultimately, we explore whether sex-specific effects occur due to: (i) intrinsic *Mtrr* deficiency by analyzing *Mtrr^gt/gt^* conceptuses or (ii) ancestral *Mtrr* deficiency by assessing several generations of wild-type progeny derived from an *Mtrr^+/gt^* maternal grandparent. Sex-specific phenotypes may contribute to our understanding of the mechanism through which folate metabolism acts during development as well as its multigenerational effects.

## Methods

### Mice and Diet

The *Mtrr^gt^* mouse line was generated by a gene-trap (gt) insertion into the *Mtrr* locus as previously described [8, 18]. C57Bl/6 mice were originally purchased from Jackson Laboratories (http://jaxmice.jax.org) and subsequently bred in-house (Supplementary Fig. S1A). C57Bl/6 mice were used as a control since the *Mtrr^gt^* mouse line was backcrossed into a C57Bl/6 genetic background [8]. *Mtrr^gt/gt^* mice were generated by mating *Mtrr^gt/gt^* mice (Supplementary Fig. S1B). The effects of *Mtrr* deficiency in the maternal grandmother (F0) were assessed by mating *Mtrr^+/gt^* females with C57Bl/6 mice (Supplementary Fig. S1C). The effects of *Mtrr* deficiency in the maternal grandfather (F0) were assessed by mating *Mtrr^+/gt^* males with C57Bl/6 females (Supplementary Fig. S1D). In both cases, their wild-type F1 daughters were mated with C57Bl/6 males to generate the wild-type F2 generation (Supplementary Fig. S1C and D). The F3 generation was produced by mating F2 wild-type females with C57Bl/6 males (Supplementary Fig. S1C and D). The F4 generation was produced by mating F3 wild-type females with C57Bl/6 males (Supplementary Fig. S1C and D). Mice were fed Rodent No. 3 breeding chow (Special Diet Services) *ad libitum* from weaning onward. This research was regulated under the Animals (Scientific Procedures) Act 1986 Amendment Regulations 2012 following ethical review by the University of Cambridge Animal Welfare and Ethical Review Body (AWERB).

### Phenotyping

Dissections were performed at embryonic (E) day 10.5 in 1× phosphate buffered saline. Noon of the day that the vaginal plug was detected was considered E0.5. Each conceptus was individually scored for phenotypes by assessing for growth defects or congenital malformations (see below for details), and photographed using a Zeiss SteReo Discovery V8 microscope with an AxioCam MRc5 camera and AxioVision 4.7.2 software (Carl Zeiss).

*Growth defects:* Growth restriction, growth enhancement, and developmental delay were phenotypes classified as growth defects. Crown-rump lengths of embryos were determined using AxioVision 4.7.2 software. An embryo with a crown-rump length that was less than or greater than 2 SDs from the mean length of C57Bl/6 embryos at E10.5 was considered to be growth restricted or growth enhanced, respectively [8]. Crown-rump length distributions were generated for each sex. Growth-restricted and growth-enhanced embryos contained a number of somite pairs that was within the normal range for E10.5 (30–39 somite pairs) according to e-Mouse Atlas Project (http://www.emouseatlas.org). Embryos that contained fewer somite pairs than was expected at E10.5 (<30 pairs) were classified as developmentally delayed, though they were otherwise phenotypically normal for that developmental stage.

*Severely affected conceptuses:* Conceptuses were also assessed for congenital malformations, which included gross placental defects (e.g. lack of chorioallantoic attachment or eccentric chorioallantoic attachment) or gross embryonic defects (e.g. failure of the neural tube to close in the spinal cord or cranial regions, pericardial edema, reversed heart looping, overall abnormal morphology, etc.) [8]. Embryos or placentas with hemorrhages or implantation sites with two or more embryos inside (i.e. twinning) were also included in this category. Some conceptuses displayed more than one severe defect.

### Genotyping

DNA samples were obtained from yolk sac or ear tissue for PCR genotyping. *Mtrr* genotypes were confirmed by PCR genotyping as previously described [8]. Sex genotypes were determined using PCR primers against *Sry* [27] and *Myog* [28], or *Ube* [29] as previously described.

### Statistical Analysis

Statistical analyses were performed using GraphPad Prism 6 software. Parametric data (e.g. fraction of females per litter, crown-rump lengths, embryo weights, placenta weights, E:P weight ratios) were analyzed using independent unpaired *t* tests. Relative risks were determined to compare males and females of each phenotypic group and pedigree, and *P* values were calculated using Fisher’s test. *P* < 0.05 was considered significant.

## Results

### *Mtrr^gt^* ^/gt^ Homozygosity Does not Cause Sexual Dimorphism of Developmental Phenotypes at E10.5

**Figure 1:**
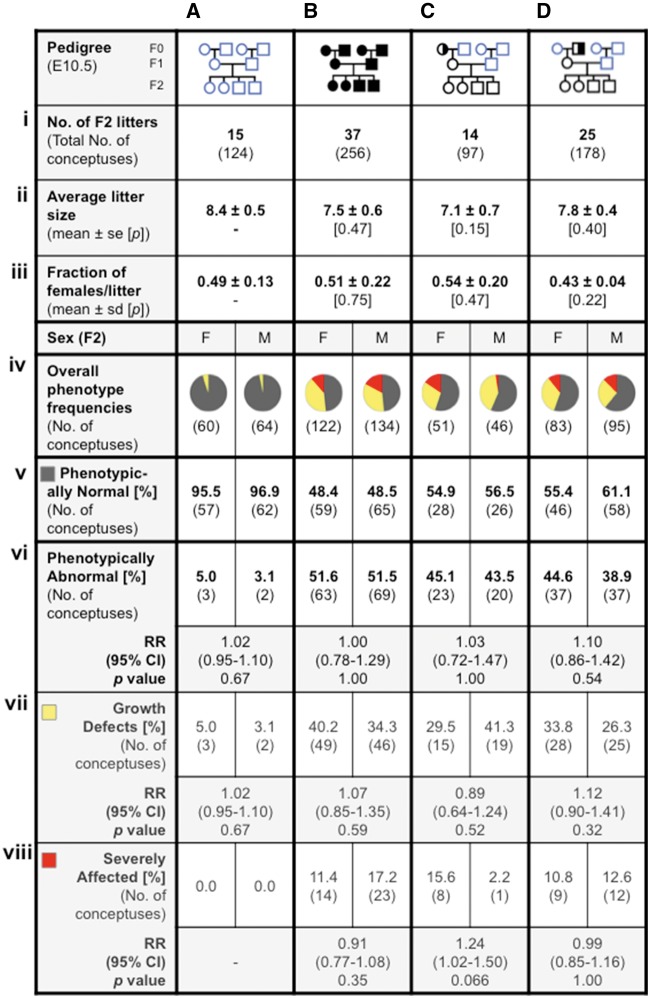
developmental phenotypes at E10.5 in *Mtrr^gt^* pedigrees are not sexually dimorphic. Conceptuses from the following pedigrees were analyzed at E10.5 in columns: (**A**) C57Bl/6 control conceptuses, (**B**) *Mtrr^gt/gt^* conceptuses, and (**C** and **D**) F2 wild-type conceptuses derived from a wild-type mother and either (**C**) an *Mtrr^+/gt^* maternal grandmother or (**D**) an *Mtrr^+/gt^* maternal grandfather. (**i**) The number of litters and total number of conceptuses assessed are indicated; (**ii**) the average litter size for each pedigree is indicated (mean ± standard error (se)); and (**iii**) average number of females per litter (±standard deviation (sd)) were calculated. In each case, independent *t* tests were performed to compare C57Bl/6 values to each experimental pedigree. (**iv–viii**) For each sex per pedigree, phenotypic frequencies were determined and represented graphically and as percentages. Two main phenotypic groups were assessed: (**v**) phenotypically normal and (**vi**) abnormal conceptuses. The percentage of phenotypically abnormal conceptuses was subdivided into two phenotypic groups: (**vii**) those with growth defects (e.g. growth enhanced, growth restricted, or developmental delayed) and (**viii**) those that were severely affected with at least one congenital malformation (e.g. neural tube, heart, and/or placenta defects). (**iv**) Pie charts show the proportion of conceptuses per sex that appeared phenotypically normal (grey), had a growth defect (yellow), or were severely affected (red). (**v–viii**) Relative risk (RR) was calculated between male and female conceptuses within each phenotype per pedigree. The 95% CI is indicated along with the *P* value as determined by a two-tailed Fisher test. F, female; M, male. Pedigree key: circle, female; square, male; blue outline, C57Bl/6 line; black outline, *Mtrr^gt^* line; white fill, *Mtrr^+/+^*; half black/half white fill, *Mtrr^+/gt^*; black fill, *Mtrr^gt/g^*.

First, to understand whether the developmental phenotypes caused by abnormal folate metabolism were sexually dimorphic, C57Bl/6 control conceptuses (*N* = 124 from 15 litters) and *Mtrr^gt/gt^* conceptuses (*N* = 256 from 37 litters) were dissected (Fig. 1A.i and B.i), scored for phenotypes at E10.5 and sexed. The average litter size and overall proportion of *Mtrr^gt/gt^* female conceptuses per litter was not significantly different compared to control litters (Fig. 1A.ii, iii and B.ii, iii). The conceptuses were then divided into two major groups: those that were phenotypically normal and those with developmental phenotypes. Phenotypic frequencies were calculated by sex for each pedigree and the relative risk of females displaying a phenotype over males were determined for statistical comparison (Fig. 1A.iv–vi and B.iv–vi). Resorptions were not accounted for due to the difficulties in obtaining fetally derived tissue from these implantation sites for sex genotyping.

In general, the sex ratio for each phenotypic group in the C57Bl/6 or *Mtrr^gt/gt^* pedigrees at E10.5 was close to the expected 1:1 ratio (Fig. 1A.v, vi and B.v, vi). For example, a similar number of *Mtrr^gt/gt^* females (51.6%) and *Mtrr^gt/gt^* males (51.5%) were identified as phenotypically abnormal (Fig. 1B.vi). The relative risk of female conceptuses developing a phenotype at E10.5 compared to males was 1.0 (95% confidence interval [CI], 0.78–1.29) (Fig. 1B.vi). The cutoff for raised relative risk was determined arbitrarily at 1.4 [30, 31]. Fisher’s statistical test also revealed no significant difference (*P* = 1.00) (Fig. 1B.vi) indicating that there is no increased risk for *Mtrr^gt/gt^* females over *Mtrr^gt/gt^*males to display any abnormal phenotype at E10.5.

Next, the broad category of phenotypically abnormal conceptuses was subdivided into growth-defective and severely affected phenotypes (Fig. 1A.vii, viii and B.vii, viii), which were previously shown to result from distinct epigenetic mechanisms [8]. Phenotypic frequencies were determined by sex and relative risks of 1.07 (95% CI, 0.85–1.35) for growth defective *Mtrr^gt/gt^* conceptuses (Fig. 1B.vii) and 0.91 (95% CI, 0.77–1.08) for severely affected *Mtrr^gt/gt^* conceptuses (Fig. 1B.viii) were determined with *P* values >0.35. Still further division of growth defective *Mtrr^gt/gt^* conceptuses into the subcategories of growth enhancement, growth restriction, and developmental delay (Supplementary Fig. S2A and B) or of severely affected *Mtrr^gt/gt^* conceptuses into the subcategories of placenta defects, heart defects, neural tube defects, hemorrhages, twinning, or overall abnormal morphology (Supplementary Fig. S3A and B) also failed to show sexual dimorphism in any phenotypic group. However, partitioning conceptuses into highly defined phenotypes leads to small sample numbers (e.g. *N* = 1–10 conceptuses per phenotype and sex, and per pedigree) and thus the data are less reliable. Regardless, the data as presented suggest the absence of any marked sexual dimorphism caused by intrinsic *Mtrr* deficiency in both broad and narrowly defined phenotypic categories at E10.5.

### Absence of Phenotypic Sexual Dimorphism at E10.5 in Wild-type Conceptuses Derived from an *Mtrr*-Deficient Maternal Grandparent

Next, we examined whether abnormal folate metabolism in either maternal grandparent caused a preferential increase of developmental phenotypes in their wild-type female grandprogeny over their male grandprogeny at E10.5. To do this, wild-type conceptuses (i.e. the F2 generation) derived from either an *Mtrr^+/gt^* maternal grandmother (*N* = 97 conceptuses from 14 litters; Fig. 1C.i) or an *Mtrr^+/gt^* maternal grandfather (*N* = 178 conceptuses from 25 litters; Fig. 1D.i) were dissected at E10.5, classified by phenotype and sex. Similar to *Mtrr^gt/gt^* litters, the average litter size and fraction of wild-type F2 female conceptuses per litter was not statistically different compared to C57Bl/6 conceptuses, regardless of whether they were derived from an *Mtrr*-deficient maternal grandmother (Fig. 1C.ii and iii) or grandfather (Fig. 1D.ii and iii).

Analysis of wild-type F2 conceptuses derived from an *Mtrr*-deficient maternal grandmother revealed no sexual dimorphism when considering all phenotypically abnormal conceptuses together based on phenotypic frequency (45.1% of females versus 43.5% of males were phenotypically abnormal) and relative risk (1.03 [95% CI, 0.72–1.47], *P* = 1.00; Fig. 1C.v and vi). Similarly, phenotypically abnormal female and male conceptuses derived from an *Mtrr*-deficient maternal grandfather were also fairly equally represented (44.6% of females versus 38.9% of males were phenotypically abnormal; relative risk: 1.10 [95% CI, 0.86–1.42], *P* = 0.54) (Fig. 1D.v and vi). Further subdivision of phenotypes by sex and pedigree, as above, also failed to reveal sexual dimorphism (Fig. 1C.vii, viii and  D.vii, viii; Supplementary Figs S2C, D and S3C, D). Overall, the data suggest that ancestral *Mtrr* deficiency does not lead to significant sexual dimorphism of developmental phenotypes in wild-type F2 conceptuses at E10.5.

Despite this, proportionately (but not significantly) more wild-type F2 female conceptuses derived from an *Mtrr*-deficient maternal grandmother were classed as severely affected compared to male littermates (15.6% [8/51] of all females displayed a severe phenotype versus only 2.2% [1/46] of all males; relative risk: 1.24 [95% CI, 1.02–1.50]; *P* = 0.066) (Fig. 1C.viii and Supplementary Fig. S3C). A larger population is required to resolve whether this is a statistically significant event but it suggests that *Mtrr* deficiency in females may differentially affect their wild-type grandprogeny, with their female grandprogeny at a greater risk for congenital malformations.

We previously demonstrated that congenital malformations are transgenerationally inherited in the *Mtrr^gt^* model since they are present at a significant frequency at E10.5 in the F3 and F4 wild-type generations derived from an *Mtrr*-deficient maternal grandparent [8]. Although growth phenotypes were also present in these litters, the frequency was not significant [8]. To determine whether sexual dimorphism exists beyond the F2 generation, we divided the wild-type F3 litters and F4 litters at E10.5 derived from each *Mtrr^+/gt^* maternal grandparent pedigree by phenotype and by sex (*N* = 53–71 conceptuses per pedigree for each generation assessed; Supplementary Figs S4–S6). Similar to *Mtrr^gt/gt^* conceptuses and F2 wild-type conceptuses, we determined that sexual dimorphism was not apparent in F3 and F4 wild-type conceptuses when considering the frequencies of overall phenotypic abnormalities at E10.5 (Supplementary Fig. S4) or specific phenotypes (Supplementary Figs S5 and S6) per pedigree.

Very few phenotypic differences were observed when comparing the effects of *Mtrr* deficiency in the maternal grandmother versus the maternal grandfather. Similar frequencies of abnormal phenotypes were present in each pedigree regardless of sex (Figs 1C and D;Supplementary Fig. S4) [this study; 32]. Some exceptions might include: (i) wild-type conceptuses from the *Mtrr^+/gt^* maternal grandfather pedigree were more likely to have a growth enhanced phenotype, particularly in the F3 generation though regardless of embryonic sex, compared to conceptuses derived from an *Mtrr^+/gt^* maternal grandmother (Supplementary Fig. S2C.iii, iv and D.iii, iv) and (ii) more female than male conceptuses from the maternal grandmother pedigree may be at an overall risk of congenital malformations compared to the maternal grandfather pedigree where there is no sex difference (Fig. 1C.viii and D.viii). Because the phenotypic frequencies are low, larger populations of each generation will need to be assessed to fully understand whether phenotypic and sexually dimorphic differences exist depending on which maternal grandparent is the carrier for the *Mtrr^gt^* allele.

### Embryonic Weights of Phenotypically Normal Conceptuses Depend on Sex and the Generational Distance from the *Mtrr*-Deficient Ancestor

**Figure 2:**
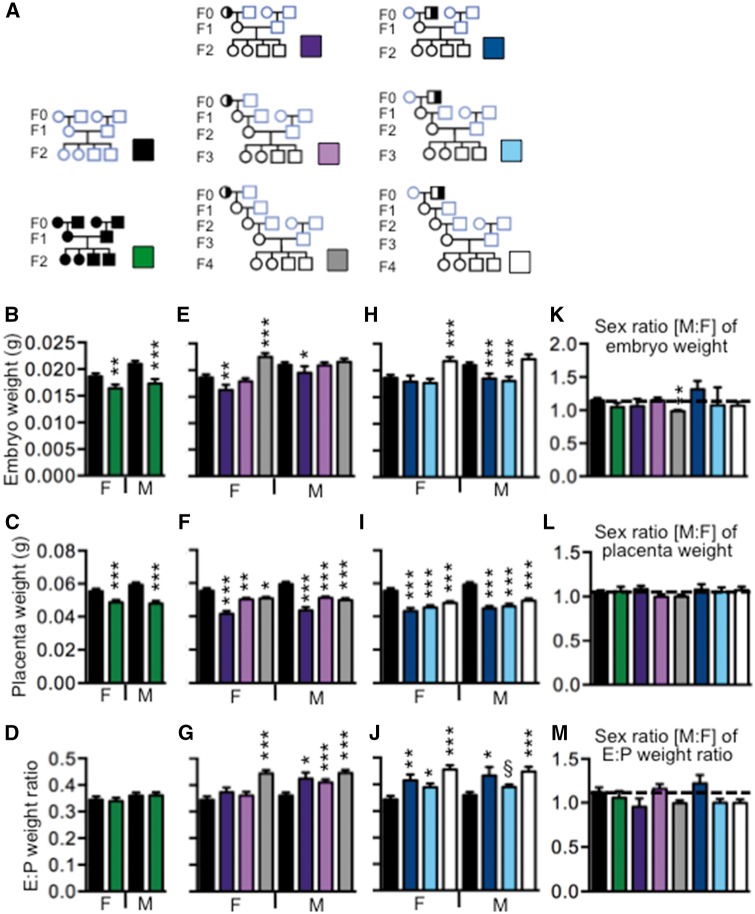
increased placental efficiency leads to an increase in embryonic weight in female conceptuses of the F4 generation. (**A**) Embryo and placental weights of phenotypically normal conceptuses from the indicated pedigrees and generations were analyzed. Conceptuses did not display any obvious developmental phenotype, had crown-rump lengths within 2 SDs of the C57Bl/6 mean crown-rump length and had 30–40 somite pairs. Data were collected from the *Mtrr^gt/gt^* pedigree (green bars; *N*=25 females and 19 males from 9 litters), the wild-type F2 generation (purple bars; *N*=18 females and 11 males from 6 litters), F3 generation (pink bars; *N*=30 female and 26 males from 8 litters), or F4 generation (dark gray bars; *N*=22 females and 29 males from 10 litters) derived from an *Mtrr^+/gt^* maternal grandmother, or the wild-type F2 generation (dark blue bars; *N*=19 females and 25 males from 9 litters), F3 generation (light blue bars; *N*=28 females and 21 males from 8 litters) or F4 generation (light gray bars; *N*=25 females and 20 males from 8 litters) derived from an *Mtrr^+/gt^* maternal grandfather. C57Bl/6 conceptuses were measured as a control (black bars; *N*=41 females and 45 males from 11 litters). (**B-J**) Graphs depicting the mean value (±se) of (B, E, H) embryo weights, (C, F, I) placenta weights, and (D, G, J) embryo:placenta (E:P) weight ratios of phenotypically normal female or male embryos at E10.5. (**K**–**M**) Male:female (M:F) ratios of each of the following parameters were determined per litter and then averaged per pedigree (±se): (K) embryo weights, (L) placenta weights, and (M) E:P weight ratios. Statistical analysis: Independent *t* tests were performed to compare C57Bl/6 mice to each experimental group by sex. *P<0.05, **P<0.01, ***P<0.005. Pedigree key: circle, female; square, male; blue outline, C57Bl/6 line; black outline, *Mtrr^gt^* line; white fill, *Mtrr^+/+^*; half black/half white fill, *Mtrr^+/gt^*; black fill, *Mtrr^gt/gt^*

Phenotypically normal conceptuses were classified by a lack of obvious congenital malformations, somite pair numbers that were within the normal range for E10.5 (i.e. 30–39 somite pairs), and crown-rump lengths that were within 2 SDs of the mean C57Bl/6 length. Previously, we showed that these so-called phenotypically normal conceptuses exposed intrinsically or ancestrally to the *Mtrr^gt^* mutation were molecularly abnormal at E10.5 since their placentas displayed widespread epigenetic instability that correlated with the dysregulation of associated genes [8]. This led us to investigate whether placenta and embryo weights in conceptuses from the phenotypically normal category of each *Mtrr* pedigree were altered compared to C57Bl/6 controls and whether the observed effects differed by sex (Fig. 2). Ultimately, this would enable us to further determine whether females or males showed an increased susceptibility to *Mtrr* deficiency with potential implications for phenotypes in later embryonic and/or adult life [1], and multigenerational adaptations to abnormal folate metabolism.

### *Mtrr^gt/^* ^g^ ^t^ *P* *edigree*

We observed that the average embryo and placenta weights of phenotypically normal female and male *Mtrr^gt/gt^* conceptuses at E10.5 were significantly reduced (*P* < 0.01) compared to C57Bl/6 controls (Fig. 2B and C) meaning that although their crown-rump lengths were within the normal range, they weighed less on average. Embryo:placenta (E:P) weight ratios were similar to controls suggesting a proportional decrease in embryo and placenta weights (Fig. 2D). Although it is remarkable that even phenotypically normal *Mtrr^gt/gt^* conceptuses at E10.5 weighed less on average, no sexual dimorphism was apparent since males and females showed a similar magnitude of reduction in each parameter (Fig. 2K–M).

### *Mtrr^gt^* *Maternal* *Grandmother Pedigree*

When ancestral *Mtrr* deficiency (up to and including the F4 generation) was considered in phenotypically normal conceptuses, pedigree-specific sexual dimorphism became apparent. First, in the *Mtrr* maternal grandmother pedigree, average placenta weights were significantly decreased in phenotypically normal females and males from generations F2 to F4 compared to C57Bl/6 controls at E10.5 (*P* < 0.05; Fig. 2F). A small placenta can have a profound effect on embryonic growth due to reduced nutrient and gas exchange. We observed that embryo growth was dependent on sex and the generational distance from the F0 *Mtrr*-deficient female. For instance, F2 wild-type female embryos (*N* = 27) weighed significantly less on average than C57Bl/6 control female embryos (*N* = 41; *P* < 0.05; Fig. 2E), an effect that was proportional with the reduced placental size (Fig. 2F) as indicated by the normal E:P weight ratio (Fig. 2G). F3 female embryo weights (*N* = 30) were within the normal range (Fig. 2E) indicating that embryo weight might be maintained by increased placental efficiency despite an E:P weight ratio that was not statistically different than controls (Fig. 2G). Remarkably, by the F4 generation, phenotypically normal wild-type female embryos (*N* = 31) weighed significantly more than C57Bl/6 control females (*P* < 0.001) despite being associated with small placentas (Fig. 2E and F). The likely cause is increased placental efficiency highlighted by an increased E:P weight ratio (Fig. 2G).

Similarly, the embryonic weights of phenotypically normal male conceptuses derived from an *Mtrr*-deficient maternal grandmother also improve over each generation but not to the extent of females from the same pedigree. Average F2 male embryo weights (*N* = 25) were low compared to C57Bl/6 male controls (*N* = 45, *P* < 0.05; Fig. 2E), while F3 and F4 male embryo weights (*N* = 26 and 20, respectively) were within the normal range. In each case, the average placental weights were reduced (Fig. 2F) and E:P ratios were high (Fig. 2G). These data suggest that all three generations of males had increased placental efficiency. However, this efficiency was only able to achieve suboptimal or optimal growth and not excessive growth as was observed in female conceptuses of the same lineage.

In general, phenotypically normal males and females from the same litter showed a similar change in embryo and placenta weights (Fig. 2K–M). The only exception to this was the F4 generation from the maternal grandmother pedigree where female embryos showed increased growth compared to males from the same litter (Fig. 2K). Altogether, these data suggest that abnormal folate metabolism in a maternal grandmother might increase placental efficiency in each successive generation of phenotypically normal wild-type grandprogeny. Ultimately, this would result in an increase of female embryonic growth and a normalization of male embryonic growth by the F4 generation.

### *Mtrr^gt^* *Maternal* *Grandfather* *Pedigree*

The effect of *Mtrr*-deficient maternal grandfathers on their phenotypically normal wild-type grandprogeny showed a comparable trend to *Mtrr*-deficient maternal grandmothers. Again, the average placental weights in the F2–F4 generations were significantly reduced regardless of sex compared to controls (*P* < 0.001; Fig. 2I). This was associated with normal embryo weights in phenotypically normal F2–F3 generation female conceptuses (*N* = 19 and 22, respectively) and increased embryo weights in the F4 generation females (*N* = 25) compared to C57Bl/6 female controls (Fig. 2H). These data suggest that within this phenotype, the F2 and F3 female placentas compensate for growth defects and, therefore, are more efficient than F2 female placentas derived from *Mtrr*-mutant maternal grandmothers. Furthermore, similar to the *Mtrr*-deficient maternal grandmother pedigree, F4 generation female conceptuses display heightened placental efficiency leading to increased embryonic growth. This was supported by an increase in E:P weight ratios (Fig. 2G and J).

On the other hand, *Mtrr* deficiency in maternal grandfathers cause their phenotypically normal male grandprogeny in the F2–F3 generations (*N* = 25 and 29, respectively) to have reduced embryonic and placental weights, and E:P weight ratios that suggest increased placental efficiency compared to C57Bl/6 male control embryos (*N* = 45, *P* < 0.001; Fig. 2J). Reduced embryonic weight in F3 males in this pedigree differs from normalized weight in F3 males in the maternal grandmother pedigree. By the F4 generation, male embryonic weight was normalized (*N* = 21, Fig. 2H) and was similar to male F4 conceptuses in the maternal grandmother pedigree. In the maternal grandfather pedigree, phenotypically normal males and females from the same litter showed a similar change in embryo and placenta weights in each generation (Fig. 2K–M).

Overall, these data suggest that male placentas are less able to adapt to ancestral abnormal folate metabolism compared to female placentas regardless of the grandparental pedigree from which they are derived. Alternatively, female placentas may be programmed to functionally overcompensate in the generations after abnormal folate metabolism or the corresponding embryos are more sensitive to nutrient availability and increase their weight more readily compared to males. Furthermore, male placentas might be less efficient when conceptuses are derived from an *Mtrr*-deficient maternal grandfather versus a maternal grandmother.

### Crown-Rump Length Distributions Differ by Sex between Generations F2–F4

**Figure 3:**
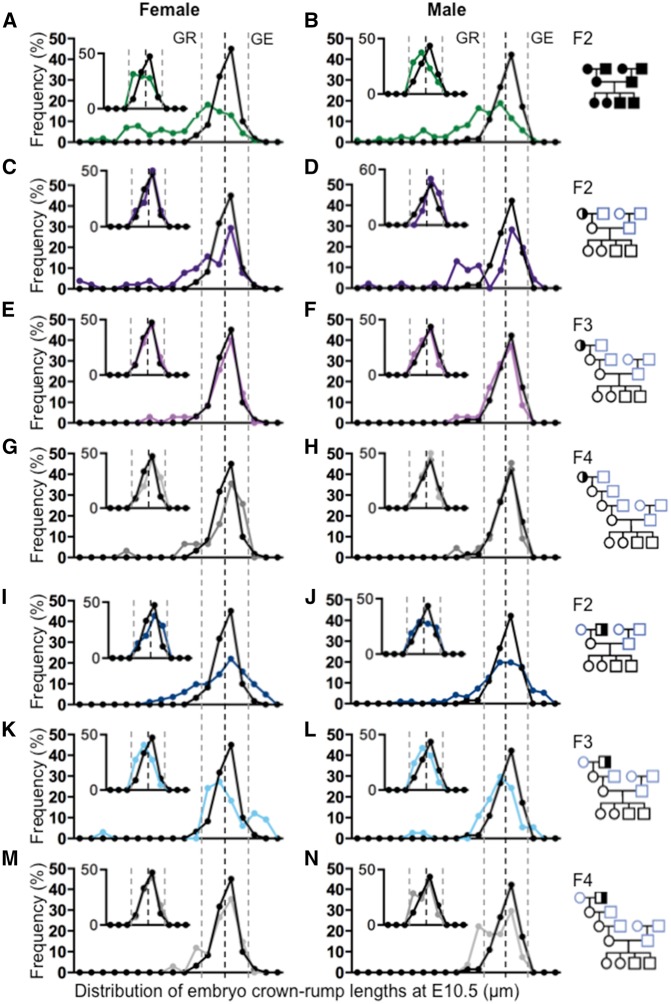
ability to normalize the crown-rump length distribution over multiple generations is sexually dimorphic. Frequency distributions curves of embryo crown-rump length at E10.5 were determined by sex in each pedigree (as indicated to the right) and compared to C57Bl/6 control distributions (black line). The larger graph in each panel represents the crown-rump length distribution of all conceptuses regardless of phenotype, whereas the smaller inset graph only represents the crown-rump length distribution of phenotypically normal embryos for the same pedigree. Female and male crown-rump length distributions were determined from the following: (**A** and **B**) *Mtrr^gt/gt^* conceptuses (green line), (**C**–**H**) wild-type conceptuses from the (C and D) F2 generation (purple line), (E and F) F3 generation (pink line), and (G and H) F4 generation (dark gray line) derived from an *Mtrr^+/gt^* maternal grandmother, and (**I**–**N**) wild-type conceptuses from the (I and J) F2 generation (dark blue line), (K and L) F3 generation (light blue line), and (M and N) F4 generation (light gray line) derived from an *Mtrr^+/gt^* maternal grandfather. Black dotted lines indicate the mean crown-rump length for C57Bl/6 control embryos. Grey dotted lines indicate two standard deviations from the control mean. Embryos with lengths that were 2 SDs below the mean are considered growth restricted (GR) (and/or displayed congenital abnormalities). Embryos with lengths that were 2 SDs over the control mean are considered growth enhanced (GE). Each point on the graph indicates the percentage of embryos within a range of crown-rump lengths equaling a standard deviation based on the C57Bl/6 growth distribution. Pedigree key: circle, female; square, male; blue outline, C57Bl/6 line; black outline, *Mtrr^gt^* line; white fill, *Mtrr^+/+^*; half black/half white fill, *Mtrr^+/gt^*; black fill, *Mtrr^gt/gt^*

Prompted by the notion that growth dynamics and placental efficiency of female and male offspring derived from an *Mtrr*-deficient ancestor may differ over multiple generations, we constructed growth distribution curves based on crown-rump lengths at E10.5 for each pedigree including all embryos regardless of developmental phenotype (Fig. 3). Overall litter sizes were generally normal in each pedigree compared to controls (Fig. 1A–D.ii and Supplementary Fig. S4A–E.ii). Litter size was not a confounding factor on embryo growth in this case since it did not negatively correlate with embryo length in any pedigree assessed at E10.5 (Supplementary Fig. S7). As expected, the growth distributions of the F2 generation from all three pedigrees were greatly disrupted compared to the C57Bl/6 curve (Fig. 3A–D and I–J). Within the *Mtrr* maternal grandmother pedigree, growth curves were largely normalized in both female and male conceptuses by the F3 generation (Fig. 3E, F, and H). However, consistent with the weight data above (Fig. 2E), the growth curve of the F4 generation female conceptuses was slightly shifted to the right (Fig. 3G) indicating increased body growth in females over time. In contrast, F3 generation female and male distribution curves derived from an *Mtrr*-deficient maternal grandfather remained abnormal (Fig. 3K and L), with a possible bimodal distribution in the female curve (Fig. 3K). By the F4 generation, the female curves were largely normalized (Fig. 3M) but the F4 male distribution curve remained bimodal and shifted to the left (Fig. 3N) indicating that a large portion of the overall F4 male growth remained reduced in size.

## Discussion

An increased risk of congenital abnormalities at birth is generally attributed to males rather than females [31]. In this study, we sought to determine whether any or all of the developmental phenotypes caused by abnormal folate metabolism in the *Mtrr^gt^* mouse model at midgestation were sexually dimorphic. We showed that *Mtrr^gt/gt^* females and males displayed an equal risk for the incidence of growth defects and congenital malformations indicating that intrinsic abnormal folate metabolism did not result in sexual dimorphism of developmental phenotypes at E10.5. Given that the *Mtrr^gt^* mouse line is a model for transgenerational inheritance, we also investigated whether a sex-specific effect on development resulted from ancestral exposure to *Mtrr* deficiency. When broadly defined, growth defects and congenital malformations were not sexually dimorphic in the F2–F4 wild-type generations derived from either *Mtrr*-deficient maternal grandparent. However, the rate at which embryonic weight was normalized over four wild-type generations was faster in female conceptuses regardless of the *Mtrr* grandparental pedigree, implying enhanced female adaptability to ancestral abnormal folate metabolism compared to males. This might be due to increased placental efficiency in females or an altered response that is intrinsic to the female embryo.

When comparing phenotypic frequencies and placental weights between the two *Mtrr* maternal grandparental pedigrees, very few differences were observed over all generations assessed. This indicates a common mechanism (most likely epigenetic) through which folate metabolism acts that leads to the multigenerational defects [8]. However, when comparing the adaptability of phenotypically normal conceptuses to ancestral abnormal folate metabolism over multiple generations, a few differences were observed when the F0 female was a carrier of the *Mtrr^gt^* allele versus when the F0 male was the carrier. First, male embryonic growth normalized one generation faster in the *Mtrr* maternal grandmother pedigree compared to the grandfather pedigree. In fact, the growth distribution for F4 wild-type males never fully recovers in the *Mtrr* maternal grandfather pedigree. Second, even though the F2 female embryo weights are worse off when their maternal grandmothers were *Mtrr*-deficient compared to their maternal grandfathers, increased embryo weights were still observed in F4 females in both pedigrees. This suggests that there may a degree of sexual dimorphism in the *Mtrr^+/gt^*F0 generation leading differential effects on their grandprogeny.

Dietary folate deficiency or defects in folate metabolism are associated with many diseases and congenital abnormalities, yet only a few studies have reported sex-specificity in offspring [21–25], and none have assessed for sex-specific effects during development. For instance, feeding rats a methyl-deficient diet at conception or prenatally differentially alters glucose homeostasis in adult male and female offspring [23, 24]. Alternatively, the incidence of C677T polymorphism in the *MTHFR* gene (important for folate metabolism) in association with urinary tract anomalies is higher in girls than boys [21]. This polymorphism along with the A1298C polymorphism in *MTHFR* gene has sex-specific associations with lung cancer risk in humans [25]. Here, while developmental phenotypes in all *Mtrr* pedigrees assessed were not sexually dimorphic at E10.5, the analysis was limited in some cases by the population size. Even though the broad phenotypic categories (i.e. growth defects or congenital malformations) were clearly not sexually dimorphic, more specific phenotypes (e.g. placenta defects, hemorrhage, or developmental delay) likely require more litters to conclusively resolve the presence of sexual dimorphism. Furthermore, assessing *Mtrr^gt/gt^* conceptuses later in development or as adults may also reveal sex-specific phenotypes.

Folate metabolism is essential for cellular methylation [14–16], and it is widely known that supplementation with folate at conception and during early pregnancy is beneficial to the health of the fetus [33–35]. During the establishment or maintenance of DNA methylation patterns in the blastocyst and postimplantation conceptus [36], it is plausible that the methylation of precise genomic regions may be responsive to abnormal folate metabolism in a sex-specific manner leading to sexually dimorphic phenotypes. Gonadal differentiation does not occur until after E10.5 [37], yet there is evidence that sexually dimorphic transcription occurs as early as the eight-cell stage together with sex-specific histone methylation patterns [12]. Even though the *Mtrr^gt^* mutation is known to cause metabolic disruption [18, 32], it is more likely that the role of MTRR plays in transmitting methyl groups for cellular methylation is responsible for the multigenerational effect that is observed. Indeed, *Mtrr^+/+^* and *Mtrr^+/gt^* mice do not have increased plasma total homocysteine compared to C57Bl/6 controls [8]. Meanwhile, intrinsic and ancestral *Mtrr* deficiency results in global DNA hypomethylation and locus-specific DNA hyper- and hypomethylation associated with altered gene expression at E10.5 [8], though earlier time points have not yet been assessed. Inter-individual differences in DNA methylation patterns caused by the *Mtrr^gt^* mutation are largely thought to be stochastic due to the wide spectrum of phenotypes observed at midgestation. However, here, the absence of sex-specific effects in *Mtrr^gt/gt^* conceptuses suggests that, at least at E10.5, sex-specific epigenetic changes are unlikely to lead to developmental phenotypes or are too subtle to detect by phenotype alone.

Environmental stressors triggering inherited epigenetic changes may ultimately lead to adaptation to environmental conditions without relying on beneficial mutations in the DNA base sequence [10]. From previous work, it is clear that adverse effects caused by *Mtrr* deficiency can persist for multiple generations through the maternal line [8]. However, whether generational adaptations to abnormal folate metabolism exist has not been assessed until now. Our data show that within the so-called phenotypically normal group of conceptuses at E10.5, placental efficiency likely increases in both female and male conceptuses in the F3-F4 generations. This may occur when placental structure or function is altered [38], such as an increase in vascularization of placental villi to increase the surface area of nutrient exchange [39] or an increase in nutrient transporter presentation [40, 41]. For example, alterations in maternal diet of a spiny mouse can lead to a male-specific decrease of placental size but an increase in expression of genes encoding nutrient transporters (e.g. *Snat2*, *Glut1*, *Glut4*) [42]. Our data that small placentas in the F3–F4 generations are associated with embryos of normal or increased weight suggest that ancestral abnormal folate metabolism might program enhanced functional development of placentas for several generations, possibly by encouraging greater metabolic efficiency of available folate through altered epigenetic regulation.

Although placental weight was decreased in females and males in the F2–F4 generations, it is remarkable that the effect of increased placental efficiency in the *Mtrr^gt^* model is not sexually equivalent. That is, despite their small size, placentas from phenotypically normal female conceptuses derived from either *Mtrr*-deficient maternal grandparent appear to function more effectively than males, causing increased embryonic growth in comparison to C57Bl/6 controls as well as previous generations. In this context, female embryos may be better able to increase placental surface area or transporter expression than males to increase their placental efficiency leading to normalization of embryonic weight by the F3 generation and an increase in embryonic weight by the F4 generation. It will be interesting to determine whether the increase in female embryo weight observed in the F4 generation is maintained in subsequent generations (e.g. F5, F6, etc.). While male embryo weight did eventually normalize, it never exceeded that of controls, which could be because males have an increased growth rate *in utero* compared to females under normal conditions [43]. Although the mechanism through which this programming occurs to alter placental efficiency is unclear, epigenetic factors are major candidates and might be regulated in a sexually dimorphic manner ultimately allowing female-specific adaptations. Possible mechanisms might include inheritance of atypical mitochondria via oocytes, and/or differential DNA/RNA methylation or non-coding RNA expression present in the germ cells that influence somatic cells of the next generation [10, 44]. As such, further experimentation of these parameters in the *Mtrr^gt^* model is required in the context of female embryonic weight gain and placental function to explore this phenomenon.

While the specific effects of intrinsic or ancestral abnormal folate metabolism on placental trophoblast development and function have not been assessed, mouse trophoblast progenitor cells express folate transporters and metabolic enzymes suggesting that folate metabolism is required for placental development [45]. Furthermore, we have observed gross placental defects (e.g. abnormal chorioallantoic attachment, placental hemorrhage) and fetal growth restriction [8], which implicates poor placental development [38, 46], in *Mtrr^gt/gt^*and F2 wild-type generation conceptuses. It will be important to determine why placental weights do not normalize in each generation subsequent to an *Mtrr*-deficient ancestor since this may have important consequences on health outcome and disease risk later in life. Also, the fact that female mice appear to adapt differently to ancestral abnormal folate metabolism than male mice may have implications for why the transgenerational effects of the *Mtrr^gt^* mutation occur only through the maternal line.

## Supplementary Material

Supplementary Figures

Supplementary Information
