# Supplementary material for: Multigenerational analysis of sex-specific phenotypic differences at midgestation caused by abnormal folate metabolism
Source: Environ Epigenet. 2017 Nov 3;3(4):dvx014. doi: 10.1093/eep/dvx014 (PMC5804557; doi:10.1093/eep/dvx014)

Supplementary Figure S1

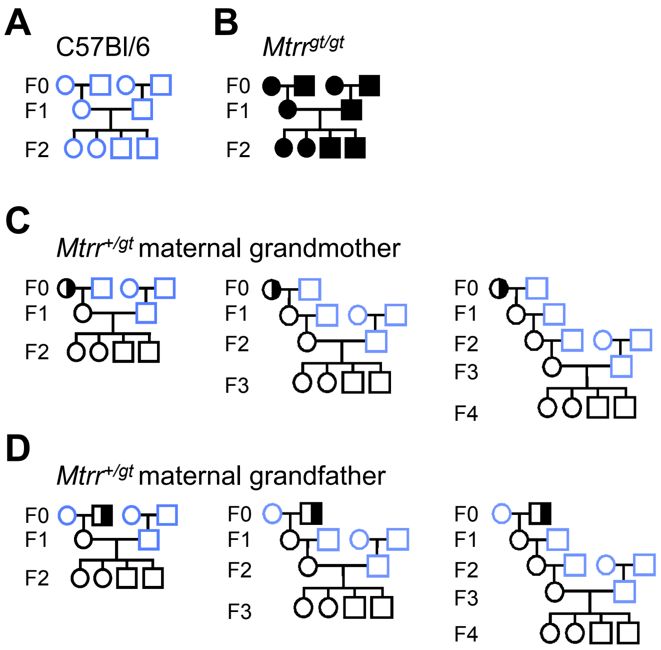

Supplementary Figure S2

		A		B		C		D	
	**Pedigree** (E10.5) F0 F1 F2	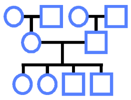		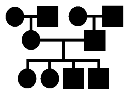		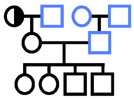		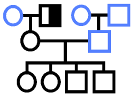	
i	**No. of F2 litters** (Total No. of conceptuses)	**15** (124)		**37** (256)		**14** (97)		**25** (178)	
	**Sex**	**F**	**M**	**F**	**M**	**F**	**M**	**F**	**M**
ii	**Growth defects [%]** (No. of conceptuses)	**5.0** (3)	**3.1** (2)	**40.2** (49)	**34.3** (46)	**29.5** (15)	**41.3** (19)	**33.8** (28)	**26.3** (25)
iii	**Proportion of growth defects**	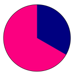	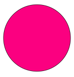	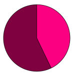	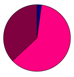	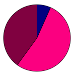	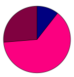	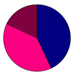	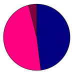
iv	**Growth enhanced [%]** (No. of conceptuses)	**33.3** (1)	**0.0**	**0.0**	**2.1** (1)	**6.7** (1)	**10.5** (2)	**42.9** (12)	**48.0** (12)
	**RR (95% CI) *p* value**	1.02 (0.98-1.05) 0.48		0.98 (0.96-1.02) 1.00		0.96 (0.85-1.09) 0.61		1.05 (0.88-1.24) 0.65	
v	**Growth restricted [%]** (No. of conceptuses)	**66.7** (2)	**100** (2)	**42.9** (21)	**60.9** (28)	**53.3** (8)	**63.2** (12)	**39.3** (11)	**48.0** (12)
	**RR (95% CI) *p* value**	1.00 (0.94-1.07) 1.00		0.95 (0.79-1.14) 0.61		0.88 (0.66-1.16) 0.44		1.03 (0.87-1.21) 0.82	
vi	**Developmen-tal delay [%]** (No. of conceptuses)	**0.0**	**0.0**	**57.1** (28)	**37.0** (17)	**40.0** (6)	**26.3** (5)	**17.9** (5)	**4.0** (1)
	**RR (95% CI) *p* value**	-		1.17 (0.97-1.40) 0.12		0.96 (0.77-1.19) 0.75		1.09 (0.99-1.20) 0.09	

Supplemental Figure S3

	A		B		C		D	
**Pedigree** (E10.5) F0 F1 F2	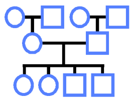		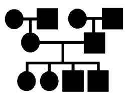		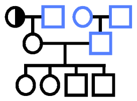		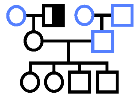	
**Sex**	**F**	**M**	**F**	**M**	**F**	**M**	**F**	**M**
i **Severely Affected [%]** (No. of conceptuses)	**0.0**	**0.0**	**11.4** (14)	**17.2** (23)	**15.6** (8)	**2.2** (1)	**10.8** (9)	**12.6** (12)
ii **Proportion of severe defects** (Total No. of severe defects)	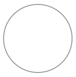 (0)	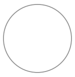 (0)	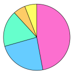 (17)	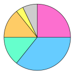 (28)	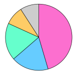 (11)	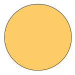 (1)	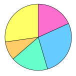 (11)	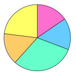 (13)
iii **Placenta defects [%]** (No. of conceptuses)	**0.0**	**0.0**	**47.1** (8)	**25.0** (7)	**45.5** (5)	**0.0**	**18.2** (2)	**15.4** (2)
**RR (95% CI)** ***p***	-		1.03 (0.91-1.15) 0.79		1.24 (1.03-1.50) 0.06		1.01 (0.94-1.09) 1.00	
iv **Heart defects [%]** (No. of conceptuses)	**0.0**	**0.0**	**23.5** (4)	**35.7** (10)	**18.2** (2)	**0.0**	**27.3** (3)	**15.4** (2)
**RR (95% CI)** ***p***	-		0.93 (0.83-1.03) 0.26		1.10 (0.97-1.24) 0.49		1.03 (0.95-1.12) 0.66	
v **Neural tube defects [%]** (No. of conceptuses)	**0.0**	**0.0**	**17.6** (3)	**14.3** (4)	**18.2** (2)	**0.0**	**18.2** (2)	**30.8** (4)
**RR (95% CI)** ***p***	-		1.02 (0.94-1.11) 0.71		1.10 (0.97-1.24) 0.49		0.98 (0.89-1.07) 0.69	
vi **Hemorrhage [%]** (No. of conceptuses)	**0.0**	**0.0**	**5.9** (1)	**14.3** (4)	**9.1** (1)	**100** (1)	**9.1** (1)	**15.4** (2)
**RR (95% CI)** ***p***	-		0.96 (0.90-1.03) 0.37		1.00 (0.87-1.14) 1.00		0.99 (0.93-1.05) 1.00	
vii **Twin/triplets [%]** (No. of conceptuses)	**0.0**	**0.0**	**5.9** (1)	**3.6** (1)	**0.0**	**0.0**	**27.3** (3)	**23.0** (3)
**RR (95% CI)** ***p***	-		1.00 (0.96-1.05) 1.00		-		1.01 (0.92-1.11) 1.00	
viii **Overall abnormal morphology [%]** (No. of conceptuses)	**0.0**	**0.0**	**0.0**	**7.1** (2)	**9.1** (1)	**0.0**	**0.0**	**0.0**
**RR (95% CI)** ***p***	-		0.99 (0.94-1.04) 1.00		1.05 (0.96-1.15) 1.00		-	

Supplementary Figure S4

		A		B		C		D		E	
	**Pedigree** (E10.5) F0 F1 F2 F3 F4	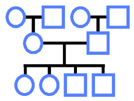		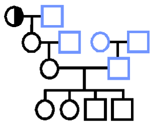		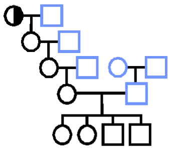		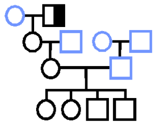		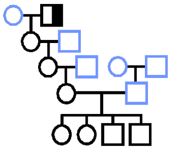	
i	**No. of litters** (Total No. of conceptuses)	**15** (124)		**10** (70)		**10** (53)		**12** (71)		**9** (61)	
ii	**Average litter size** (mean ± se [*p*])	**8.4 ± 0.5** -		**8.7 ± 0.3** [0.630]		**9.7 ± 0.8** [0.036]*		**7.8 ± 0.3** [0.279]		**9.6 ± 0.4** [0.104]	
iii	**Fraction of females/litter** (mean ± sd [p])	**0.49 ± 0.13** -		**0.48 ± 0.24** [0.918]		**0.65 ± 0.20** [0.070]		**0.47 ± 0.30** [0.863]		**0.60 ± 0.20** [0.188]	
	**Sex**	**F**	**M**	**F**	**M**	**F**	**M**	**F**	**M**	**F**	**M**
iv	**Proportion of phenotypes** (No. of conceptuses)	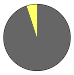 (60)	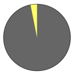 (64)	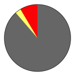 (35)	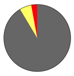 (35)	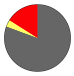 (31)	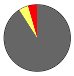 (22)	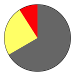 (33)	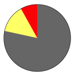 (37)	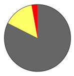 (34)	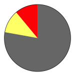 (27)
v	**Phenotypically Normal** (No. of conceptuses)	**95.0** (57)	**96.9** (62)	**88.6** (31)	**91.4** (32)	**80.6** (25)	**90.9** (20)	**63.6** (22)	**75.5** (29)	**82.4** (28)	**77.8** (21)
vi	**Phenotypically Abnormal** (No. of conceptuses)	**5.0** (3)	**3.1** (2)	**11.4** (4)	**8.6** (3)	**19.4** (6)	**9.1** (2)	**36.4** (11)	**24.3** (8)	**17.6** (6)	**22.2** (6)
	**RR (95% CI)** ***p***	**1.02 (0.95-1.10) 0.67**		**1.03 (0.88-1.21) 1.00**		**1.13 (0.91-1.40) 0.45**		**1.18 (0.88-1.58) 0.30**		**0.94 (0.73-1.22) 0.75**	
vii	**Growth Defects** (No. of conceptuses)	**5.0** (3)	**3.1** (2)	**2.8** (1)	**5.7** (2)	**3.3** (1)	**4.5** (1)	**27.3** (8)	**16.2** (5)	**14.7** (5)	**11.1** (3)
	**RR (95% CI)** ***p***	**1.02 (0.95-1.10) 0.67**		**0.97 (0.88-1.08) 1.00**		**0.99 (0.88-1.12) 1.00**		**1.16 (0.90-1.50) 0.35**		**1.03 (0.84-1.27) 1.00**	
viii	**Severely Affected** (No. of conceptuses)	**0.0**	**0.0**	**8.6** (3)	**2.9** (1)	**16.1** (5)	**4.5** (1)	**9.1** (3)	**8.1** (3)	**2.9** (1)	**11.1** (3)
	**RR (95% CI)** ***p***	-		**1.06 (0.94-1.20) 0.61**		**1.14 (0.95-1.38) 0.38**		**1.03 (0.86-1.24) 1.00**		**0.91 (0.77-1.07) 0.32**	

Supplementary Figure S5

	A		B		C		D		E	
**Pedigree** (E10.5) F0 F1 F2 F3 F4	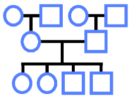		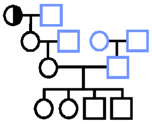		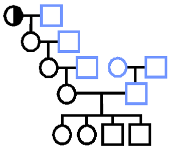		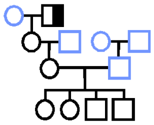		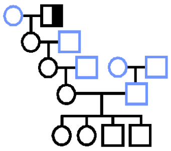	
i **No. of litters** (Total No. of conceptuses)	**15** (124)		**10** (70)		**10** (53)		**12** (71)		**9** (61)	
**Sex**	**F**	**M**	**F**	**M**	**F**	**M**	**F**	**M**	**F**	**M**
ii **Growth Defects [%]** (No. of conceptuses)	**5.0** (3)	**3.1** (2)	**2.8** (1)	**5.7** (2)	**3.3** (1)	**4.5** (1)	**27.3** (8)	**16.2** (5)	**14.7** (5)	**11.1** (3)
iii **Proportion of growth defects**	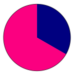	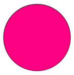	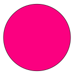	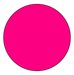	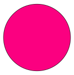	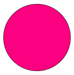	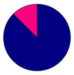	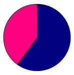	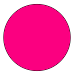	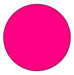
iv **Growth enhanced [%]** (No. of conceptuses)	**33.3** (1)	**0.0**	**0.0**	**0.0**	**0.0**	**0.0**	**87.5** (7)	**60.0** (3)	**0.0**	**0.0**
**RR (95% CI)** ***p***	**1.02 (0.98-1.05) 0.48**		**-**		**-**		**1.23 (0.98-1.55) 0.08**		**-**	
v **Growth restricted [%]** (No. of conceptuses)	**66.7** (2)	**100** (2)	**100** (1)	**100** (2)	**100** (1)	**100** (1)	**12.5** (1)	**40.0** (2)	**100** (5)	**100** (3)
**RR (95% CI)** ***p***	**1.00 (0.94-1.07) 1.00**		**0.97 (0.88-1.08) 1.00**		**0.99 (0.88-1.12) 1.00**		**0.95 (0.82-1.09) 0.63**		**0.99 (0.84-1.27) 1.00**	
vi **Developmental delay [%]** (No. of conceptuses)	**0.0**	**0.0**	**0.0**	**0.0**	**0.0**	**0.0**	**0.0**	**0.0**	**0.0**	**0.0**
**RR, (95% CI), *p***	**-**		**-**		**-**		**-**		**-**	

Supplementary Figure S6

	A		B		C		D		E	
**Pedigree** (E10.5) F0 F1 F2 F3 F4	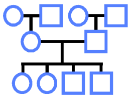		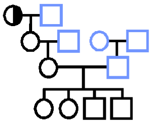		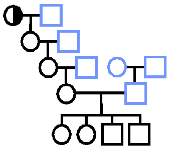		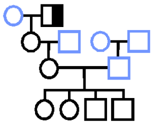		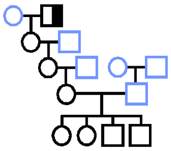	
**Sex**	**F**	**M**	**F**	**M**	**F**	**M**	**F**	**M**	**F**	**M**
i **Severely Affected [%]** (No. of conceptuses)	**0.0**	**0.0**	**8.6** (3)	**2.9** (1)	**16.1** (5)	**4.5** (1)	**9.1** (3)	**8.1** (3)	**2.9** (1)	**11.1** (3)
ii **Proportion of severe defects** (Total No. of severe defects)	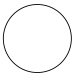 (0)	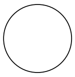 (0)	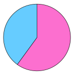 (5)	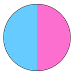 (2)	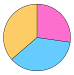 (11)	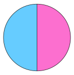 (2)	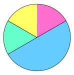 (6)	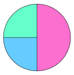 (4)	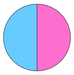 (2)	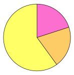 (5)
iii **Placenta defects [%]** (No. of conceptuses)	**0.0**	**0.0**	**60.0** (3)	**50.0** (1)	**27.2** (3)	**50.0** (1)	**16.7** (1)	**50.0** (2)	**50.0** (1)	**0.0**
**RR (95% CI)** ***p***	**-**		**1.06 (0.94-1.20) 0.61**		**1.05 (0.92-1.20) 0.64**		**0.98 (0.86-1.11) 1.00**		**1.03 (0.97-1.09) 1.00**	
iv **Heart defects [%]** (No. of conceptuses)	**0.0**	**0.0**	**40.0** (2)	**50.0** (1)	**36.4** (4)	**50.0** (1)	**50.0** (3)	**25.0** (1)	**50.0** (1)	**20.0** (1)
**RR (95% CI)** ***p***	**-**		**1.03 (0.93-1.15) 1.00**		**1.08 (0.93-1.25) 0.64**		**1.10 (0.93-1.29) 0.32**		**0.99 (0.91-1.09) 1.00**	
v **Neural tube defects [%]** (No. of conceptuses)	**0.0**	**0.0**	**0.0**	**0.0**	**0.0**	**0.0**	**16.7** (1)	**25.0** (1)	**0.0**	**0.0**
**RR (95% CI)** ***p***	**-**		**-**		**-**		**1.01 (0.91-1.13) 1.00**		**-**	
vi **Hemorrhage [%]** (No. of conceptuses)	**0.0**	**0.0**	**0.0**	**0.0**	**36.4** (4)	**0.0**	**0.0**	**0.0**	**0.0**	**20.0** (1)
**RR (95% CI)** ***p***	**-**		**-**		**1.13 (1.00-1.27) 0.15**		**-**		**0.96 (0.90-1.04) 0.45**	
vii **Twins/triplets [%]** (No. of conceptuses)	**0.0**	**0.0**	**0.0**	**0.0**	**0.0**	**0.0**	**16.7** (1)	**0.0**	**0.0**	**60.0** (3)
**RR (95, CI)** ***p***	**-**		**-**		**-**		**1.05 (0.96-1.14) 0.44**		**0.90 (0.80-1.01) 0.10**	
viii **Overall abnormal morphology [%]** (No. of conceptuses)	**0.0**	**0.0**	**0.0**	**0.0**	**0.0**	**0.0**	**0.0**	**0.0**	**0.0**	**0.0**
**RR, *p***	**-**		**-**		**-**		**-**		**-**	

Supplementary Figure S7

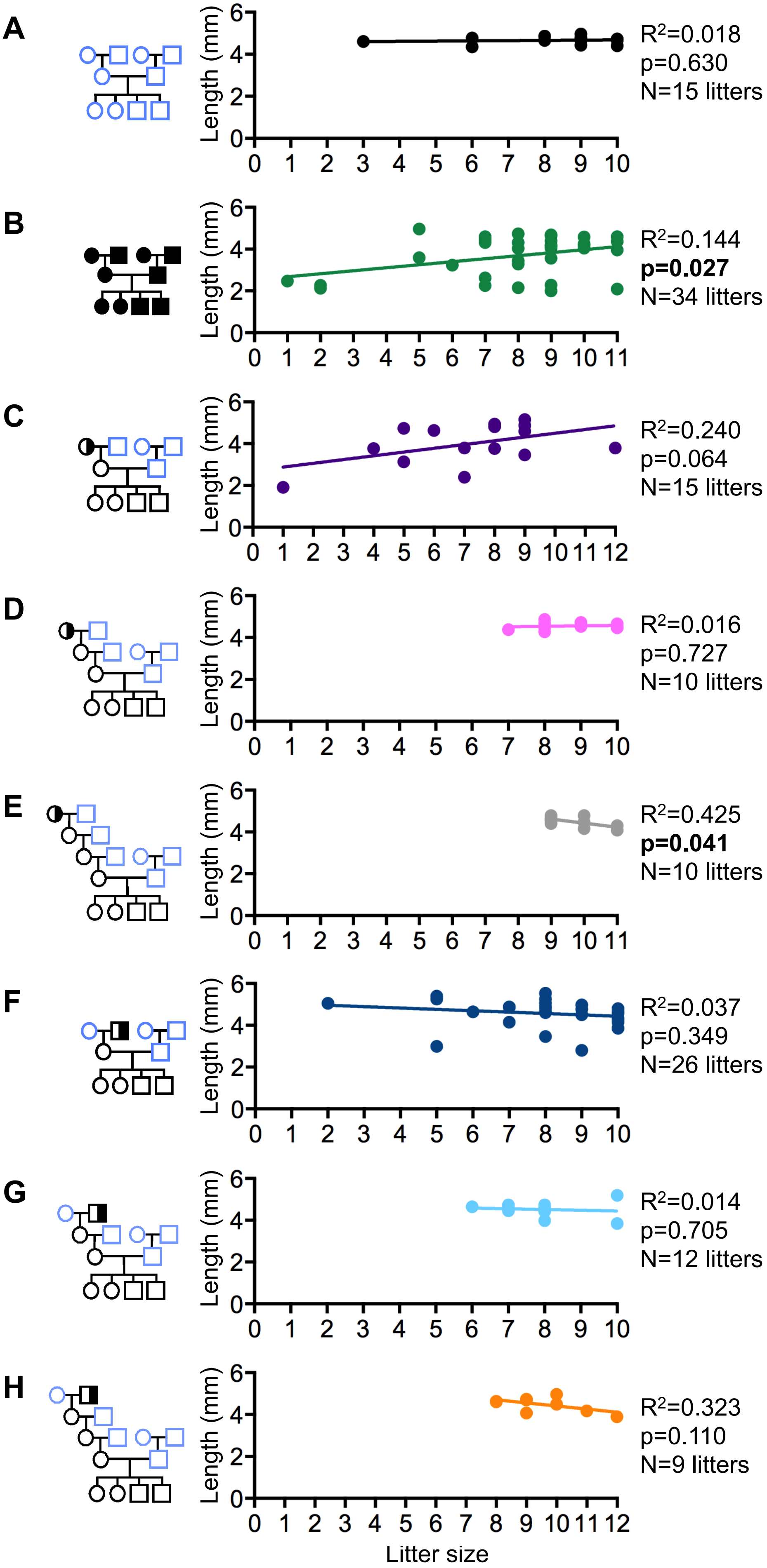

Supplement: Supplementary Figures [file dvx014_supp_figures.pdf]
