# Supplementary material for: Multigenerational analysis of sex-specific phenotypic differences at midgestation caused by abnormal folate metabolism
Source: Environ Epigenet. 2017 Nov 3;3(4):dvx014. doi: 10.1093/eep/dvx014 (PMC5804557; doi:10.1093/eep/dvx014)
Supplement: Supplementary Information [file dvx014_supp_information.docx]

**Supplementary figure legends**

**Figure S1.** *Mtrr^gt^* pedigrees used to identify whether intrinsic or ancestral exposure to abnormal folate metabolism causes sexually dimorphic phenotypes at E10.5.

(**A**) The C57Bl/6 control pedigree. (**B**) Intrinsic effect of abnormal folate metabolism was determined using *Mtrr^gt/gt^* conceptuses derived from *Mtrr^gt/gt^* intercrosses. (**C-D**) The consequence of ancestral exposure to abnormal folate metabolism was determined by analyzing the effect of *Mtrr* heterozygosity in (**C**) the maternal grandmother or (**D**) the maternal grandfather on wildtype conceptuses in the F2, F3 and F4 generation. Pedigree key: Circle, female; squares, male; blue outline, C57Bl/6; black outline, *Mtrr* line; white fill, *Mtrr^+/+^*; half white/half black, *Mtrr^+/gt^*; black fill, *Mtrr^gt/gt^*.

**Figure S2.** Sexual dimorphism of growth defects is unlikely in the *Mtrr^gt/gt^* and *Mtrr*-deficient maternal grandparental pedigrees.

The frequency of growth phenotypes (e.g., growth enhancement, growth restriction, and developmental delay) was analyzed in the following conceptuses at E10.5 in columns: (**A**) C57Bl/6 control conceptuses, (**B**) *Mtrr^gt/gt^* conceptuses, (**C**) F2 wildtype conceptuses derived from an *Mtrr^+/gt^* maternal grandmother, or (**D**) F2 wildtype conceptuses derived from an *Mtrr^+/gt^* maternal grandfather. (**i**) The number of litters and total number of conceptuses assessed per pedigree are specified. (**ii**) The percentage of conceptuses with any type of growth defect is indicated followed by the number of conceptuses this represents. (**iii-vi**) For each sex per pedigree, the frequency of specific growth phenotypes was determined and represented graphically and as percentages. (**iii**) The pie charts show the percentage of conceptuses with growth enhancement (blue), growth restriction (light pink), or developmentally delay (dark pink) within the broad category of growth defects. (**iv-vi**) These frequencies are also represented as percentages followed by the number of conceptuses with each growth phenotype in brackets. (**iv**) Growth enhancement and (**v**) growth restriction were determined by crown-rump lengths that were greater or less than two standard deviations, respectively, from the C57Bl/6 mean crown-rump length. (**vi**) Developmental delay was determined when embryos had <29 somite pairs but otherwise looked normal. Relative risk (RR) was calculated between male and female conceptuses within each phenotype per pedigree. The 95% confidence interval (CI) is indicated along with the *p* value as determined by a two-tailed Fisher test. *p<0.05, **p<0.01, ***p<0.005. F, female; M, male. Pedigree key: Circle, female; squares, male; blue outline, C57Bl/6; black outline, *Mtrr* line; white fill, *Mtrr^+/+^*; half white/half black, *Mtrr^+/gt^*; black fill, *Mtrr^gt/gt^*.

**Figure S3.** Sexual dimorphism of severe defects is unlikely in the *Mtrr^gt/gt^* and *Mtrr*-deficient maternal grandparental pedigrees.

The frequency of severe abnormalities (e.g., placental defects, heart defects, neural tube defects, hemorrhages, twinning, or overall abnormal morphology) was assessed in the following conceptuses at E10.5 in columns: (**A**) C57Bl/6 control conceptuses, (**B**) *Mtrr^gt/gt^* conceptuses, (**C**) F2 wildtype conceptuses derived from an *Mtrr^+/gt^* maternal grandmother, or (**D**) F2 wildtype conceptuses derived from an *Mtrr^+/gt^* maternal grandfather. (**i**) For each sex per pedigree, the percentage of conceptuses with severe defects out of the total number of conceptuses is indicated followed by number of conceptuses that this represents in brackets. (**ii-viii**) Phenotypic frequencies were determined for each type of severe defect and represented graphically and as percentages. (**ii**) The pie charts show the proportion of conceptuses with placenta defects (pink), heart defects (blue), neural tube defects (green), hemorrhage in placenta or embryo (orange), multiple conceptuses per implantation site (i.e., twins/triplets; yellow), or overall abnormal morphology in the embryo (gray) within the broad category of severe defects. (**iii-viii**) This data is also represented as percentages followed by the number of conceptuses with each phenotype in brackets. Note that a conceptus may display more than one severe defect, which is why the number of severely affected conceptuses in (**i**) does not equal the total number of severe defects observed in (**ii**). Relative risk (RR) was calculated between male and female conceptuses within each phenotype per pedigree. The 95% confidence interval (CI) is indicated along with the *p* value as determined by a two-tailed Fisher test. *p<0.05, **p<0.01, ***p<0.005. F, female; M, male. Pedigree key: Circle, female; squares, male; blue outline, C57Bl/6; black outline, *Mtrr* line; white fill, *Mtrr^+/+^*; half white/half black, *Mtrr^+/gt^*; black fill, *Mtrr^gt/gt^*.

**Figure S4.** Broadly classed developmental phenotypes at E10.5 in wildtype conceptuses in F3 and F4 generations derived from an *Mtrr^+/gt^* maternal grandparent are unlikely to be sexually dimorphic.

Conceptuses from the following pedigrees were analyzed at E10.5 in columns: (**A**) C57Bl/6 control conceptuses, (**B**) *Mtrr^gt/gt^* conceptuses, or (**C-D**) wildtype conceptuses of the F2 generation derived from (**C**) an *Mtrr^+/gt^* maternal grandmother or (**D**) an *Mtrr^+/gt^* maternal grandfather. (**i**) The number of litters and total number of conceptuses assessed are indicated. (**ii**) The average litter sizes for each pedigree (mean ± standard error (se)) and (**iii**) the average number of females per litter (± sd) were calculated. In each case, independent t tests were performed to compare C57Bl/6 values to each experimental pedigree. (**iv-viii**) For each sex per pedigree, phenotypic frequencies were determined and represented graphically and as percentages. Two main phenotypic groups were assessed: (**v**) phenotypically normal and (**vi**) abnormal conceptuses. The percentage of phenotypically abnormal conceptuses was subdivided into two phenotypic groups: (**vii**) those with growth defects (e.g., growth enhanced, growth restricted or developmental delayed) and (**viii**) those that were severely affected with at least one congenital malformation (e.g., neural tube, heart, and/or placenta defects, etc.). (**iv**) Pie charts display the proportion of conceptuses per sex that appeared phenotypically normal (grey), had a growth defect (yellow) or were severely affected (red). Relative risk (RR) was calculated between male and female conceptuses within each phenotype per pedigree. The 95% confidence interval (CI) is indicated along with the *p* value as determined by a two-tailed Fisher test. F, female; M, male. Pedigree key: circle, female; square, male; blue outline, C57Bl/6 line; black outline, *Mtrr^gt^* line; white fill, *Mtrr^+/+^*; half black/half white fill, *Mtrr^+/gt^*.

**Figure S5.** Sexual dimorphism of growth defects is unlikely in the F3 or F4 generations derived from an *Mtrr*-deficient maternal grandparent.

Growth phenotype frequency was analyzed in the following conceptuses at E10.5 in columns: (**A**) C57Bl/6 control conceptuses, (**B**) the F3 or (**C**) the F4 generation of wildtype conceptuses derived from an *Mtrr^+/gt^* maternal grandmother, or (**D**) the F3 or (**E**) the F4 generation of wildtype conceptuses derived from an *Mtrr^+/gt^* maternal grandfather. (**i**) The number of litters and total number of conceptuses assessed per pedigree are specified. (**ii**) For each sex per pedigree, phenotypic frequencies were determined and represented graphically and as percentages. (**iii**) The pie charts show the percentage of conceptuses with growth enhancement (blue), growth restriction (light pink), or developmentally delay (dark pink) within the broad category of growth defects. (**iv-vi**) These frequencies are also represented as percentages followed by the number of conceptuses with each growth phenotype in brackets. (**iv**) Growth enhancement and (**v**) growth restriction were determined by crown-rump lengths that were greater or less than two standard deviations, respectively, from the C57Bl/6 mean crown-rump length. (**vi**) Developmental delay was determined when embryos had <29 somite pairs but otherwise looked normal. Relative risk (RR) was calculated between male and female conceptuses within each phenotype per pedigree. The 95% confidence interval (CI) is indicated along with the *p* value as determined by a two-tailed Fisher test. *p<0.05, **p<0.01, ***p<0.005. F, female; M, male. Pedigree key: Circle, female; squares, male; blue outline, C57Bl/6; black outline, *Mtrr* line; white fill, *Mtrr^+/+^*; half white/half black, *Mtrr^+/gt^*.

**Figure S6.** Severe defects are unlikely to display sexual dimorphism in the F3 and F4 generations derived from an *Mtrr^+/gt^* maternal grandparent.

The frequency of severe defects was assessed in the following conceptuses at E10.5 in columns: (**A**) C57Bl/6 control conceptuses, (**B-C**) wildtype conceptuses in the (**B**) F3 generation or (**C**) F4 generation derived from an *Mtrr^+/gt^* maternal grandmother, or (**D-E**) wildtype conceptuses in the (**D**) F3 generation or (**E**) F4 generation derived from an *Mtrr^+/gt^* maternal grandfather. (**i**) The percentage of conceptuses with severe defects is indicated followed by number of conceptuses that this represents in brackets for each sex per pedigree. (**ii-viii**) Phenotypic frequencies for each defect were determined and represented graphically and as percentages. (**ii**) The pie charts show the proportion of conceptuses with placenta defects (pink), heart defects (blue), neural tube defects (green), hemorrhage in placenta or embryo (orange), multiple conceptuses per implantation site (i.e., twins/triplets; yellow), or overall abnormal morphology in the embryo (gray) within the broad category of severe defects. (**iii-viii**) This data is also represented as percentages followed by the number of conceptuses with each phenotype in brackets. Note that a conceptus may display more than one severe defect, which is why the number of conceptuses with severe defects in (**i**) does not equal the total number of severe defects observed in (**ii**). Relative risk (RR) was calculated between male and female conceptuses within each phenotype per pedigree. The 95% confidence interval (CI) is indicated along with the *p* value as determined by a two-tailed Fisher test. *p<0.05, **p<0.01, ***p<0.005. F, female; M, male. Pedigree key: Circle, female; squares, male; blue outline, C57Bl/6; black outline, *Mtrr* line; white fill, *Mtrr^+/+^*; half white/half black, *Mtrr^+/gt^*.

**Figure S7.** Alterations in embryo crown-rump length at E10.5 caused by direct or ancestral *Mtrr* deficiency do not correlate with litter size.

Graphs showing litter sizes plotted against the average embryo crown-rump lengths of each individual litters at E10.5 were prepared for the following pedigrees and generations: (**A**) C57Bl/6 controls, (**B**) *Mtrr^gt/gt^*, (**C-E**) wildtype embryos derived from an *Mtrr^+/gt^* maternal grandmother and representing the (**C**) F2, (**D**) F3, and (**E**) F4 generations, and (**F-H**) wildtype embryos derived from an *Mtrr^+/gt^* maternal grandfather and representing the (**F**) F2, (**G**) F3, and (**H**) F4 generations. Each graph was assessed by a linear regression model to obtain an R^2^ value. An R^2^ value close to 1 indicates a positive or negative correlation. The p value indicates whether the slope of the line is significantly non-zero. The number of litters assessed per pedigree and generation is also indicated.
